# Costs Associated with Intravenous Cancer Therapy Administration in Patients with Metastatic Soft Tissue Sarcoma in a US Population

**DOI:** 10.1155/2013/947413

**Published:** 2013-12-23

**Authors:** Mei Sheng Duh, Michelle D. Hackshaw, Jasmina I. Ivanova, Gregory Kruse, Lesley-Ann N. Miller, Patrick Lefebvre, Paul Karner, Bruce Wong

**Affiliations:** ^1^Analysis Group, Inc., 111 Huntington Avenue, Tenth Floor, Boston, MA 02199, USA; ^2^GlaxoSmithKline, Philadelphia, PA 19102, USA; ^3^University of Pennsylvania, Philadelphia, PA 19104, USA; ^4^Bruce Wong and Associates, Inc., Wayne, PA 19087, USA

## Abstract

*Background*. The most common chemotherapies in metastatic soft tissue sarcoma (mSTS) require intravenous (IV) administration. This often requires patients to make multiple outpatient visits per chemotherapy cycle, possibly impeding patients' daily activities and increasing caregiver burden and medical costs. This study investigated costs associated with IV cancer therapy administration in mSTS from the payer perspective of the health care system. *Patients and Methods*. From the Experian Healthcare database, 1,228 mSTS patients were selected. Data were analyzed on outpatient visits during 2005–2012 involving IV cancer therapy administration. Costs were estimated on a per patient per visit (PPPV) and per patient per month (PPPM) basis. *Results*. The mean (median) cost of IV therapy was $2,427 ($1,532) PPPV and $5,468 ($4,310) PPPM, of which approximately 60% was IV drug costs. IV administration costs averaged $399 PPPV and $900 PPPM, representing 16.5% of total visit costs. Anthracycline and alkylating-agents-based therapies had the highest PPPV and PPPM IV administration costs, respectively (mean $479 and $1,336, resp.). Patients with managed care insurance had the highest IV administration costs (mean $504 PPPV; $1,120 PPPM). *Conclusions*. IV administration costs constitute a considerable proportion of the total costs of receiving an IV cancer therapy to treat mSTS.

## 1. Introduction

Soft tissue sarcomas (STS) are a rare, complex group of childhood and adult neoplasms with differentiation towards mesenchymal tissue, which may arise almost anywhere in the body [1]. STS account for approximately 1% of malignant tumours in adults and 2% of total cancer mortality [2]. It is estimated that in 2012 approximately 10,280 people were diagnosed with STS in the United States (USA) [3]. STS exhibit remarkable histologic diversity and consist of a heterogeneous group of tumours with over 50 subtypes [2]. In a recent study of 17,364 cases of STS, malignant fibrous histiocytoma (24.1%), leiomyosarcoma (14.8%), sarcoma (12.8%), and myxoid liposarcoma (5.9%) were the most prevalent histological subtypes [4]. Although local control can be obtained through the use of surgery and radiotherapy, in patients who experience recurrence at distant sites (~50% of all patients), >90% will ultimately die of this malignancy [5]. The five-year survival rate for patients with advanced/metastatic disease is also low (e.g., 8% in patients with lung metastasis [6]).

The most commonly used chemotherapies in metastatic soft tissue sarcoma (mSTS) are intravenously (IV) administered agents [7]. Although the cost burden of IV cancer therapy (e.g., cost of IV administration and the cost of IV drugs) has been studied for specific cancer types (e.g., breast and small cell lung cancer) [8, 9] and other conditions, such as rheumatoid arthritis [10], no study to date has assessed the costs associated with IV cancer therapy administration in a mSTS population.

This study reports estimates of the total and component costs associated with IV cancer therapy administration in patients with mSTS from the payer perspective of the US health care system, based on an analysis of claims data from a large contract and claims management system.

## 2. Methods

### 2.1. Data Sources

Data for this study were obtained from the Experian Healthcare (Experian) database, which maintains a contract and claims management system that supports 350 general/oncology clinics in the US. The database contains a complete history of diagnoses (ICD-9-CM codes), procedures, and drug therapies received by both publicly and privately insured patients within the clinics, as well as patient demographics (e.g., age, gender, and geographic region) and insurance type (e.g., managed care, indemnity, Medicare, and Medicaid). For every patient clinic visit, Experian records the service dates, total charged, total contracted payments, and total allowed, with individual services, procedures, and drugs broken out by line item (Current Procedural Terminology, Fourth Edition [CPT-4] and Healthcare Common Procedure Coding System [HCPCS] codes). The Experian dataset used in this study covers the period from January 1, 2005, to April 30, 2012.

### 2.2. Study Design

This study employed a retrospective, longitudinal cohort design. The index date was defined as the date of the first IV cancer therapy infusion for mSTS treatment. Therapy windows were calculated as a patient-drug combination for the purposes of reporting study outcomes on a per month basis. The observation period for each patient-drug window begins at the index date and ends with either the last IV cancer therapy administration (if the patient only remains on one therapy) or the last visit of that IV cancer therapy before a patient switches to a different IV cancer therapy, plus a therapeutic effect. The therapeutic effect for patients that do not switch therapies is the average interval between administrations across the entire dataset for that particular IV cancer therapy. For patients who received a single IV therapy, the observation period length was calculated as the length of the therapy plus the average interval across the entire dataset for that particular IV cancer therapy. For patients that switch IV cancer therapies, the therapeutic effect is defined as the minimum of either the time between the last visit of that therapy and the start of the new therapy or average therapy interval across the entire dataset.

### 2.3. Study Population

**Figure 1:**
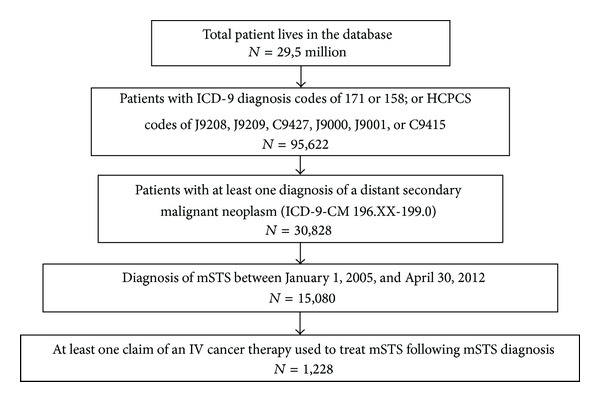
Sample selection.

The selection of patients in the study sample is depicted in Figure 1. The population of patients with mSTS receiving IV therapy was determined by a claims algorithm [11] that required patients to have (1) at least one diagnosis of a distant secondary malignant neoplasm (ICD-9-CM 196.XX-199.0); (2) a diagnosis of mSTS between January 1, 2005, and April 30, 2012; and (3) at least one claim of an IV cancer therapy used to treat mSTS (defined according to NCCN treatment guidelines [7]) following mSTS diagnosis. (For the purposes of this study, IV cancer therapies used to treat mSTS included actinomycinD, bevacizumab, carboplatin, cisplatin, cyclophosphamide, dacarbazine, docetaxel, doxorubicin, epirubicin, etoposide, gemcitabine, ifosfamide, interferon, irinotecan, mesna, oxaliplatin, paclitaxel, temozolomide, topotecan, vincristine, and vinorelbine.) Diagnosis of mSTS was defined as at least two medical claims with an ICD-9-CM diagnosis code of 171, or the following combination of IV cancer therapies and other selected ICD-9-CM diagnoses:

- a combination therapy of ifosfamide (HCPCS: J9208, J9209, C9427) and doxorubicin or liposomal doxorubicin (HCPCS: J9000, J9001, C9415) (both agents to be administered within 30 days), except if also diagnosed with osteosarcoma (ICD-9-CM: 170), nasopharyngeal carcinoma (ICD-9-CM: 147), lung cancer (ICD-9-CM:162.3-162.9), uterine cancer (ICD-9-CM: 179, 180, 182), breast cancer (ICD-9-CM:174), prostate cancer (ICD-9-CM: 185), renal cancer (ICD-9-CM: 189), or malignant neoplasm of lymphatic and hematopoietic tissue (ICD-9-CM: 200-208) at any time, or
- at least 1 medical claim for retroperitoneal or peritoneal cancer (ICD-9-CM: 158), except if also diagnosed with renal cell carcinoma (ICD-9-CM: 189.0, 198.0), transitional cell carcinoma (ICD-9-CM: 189.1, 189.2), mesothelioma (ICD-9-CM: 163), gastrointestinal stromal tumors (ICD-9-CM: 159.0, 159.8, 159.9), uterine cancer (ICD-9-CM: 179, 180, 182), ovarian cancer (ICD-9-CM: 183), or other and unspecified female genital-organ cancer (ICD-9-CM: 184) at any time.

### 2.4. Treatment Costs and IV Administration Related Costs

Treatment costs were estimated using the contracted allowed payment for a claim rather than the practice charges, based on adjudication of the claim by the patient's third-party insurance plan. A contracted payment is defined as the amount that the provider is eligible to receive from all parties, including primary and secondary payers and the patient, based on the contractual agreement with the payer. Because the contracted payment represents the actual payment to providers from payers, it depicts a more accurate and detailed view of the true economic burden to payers of IV cancer therapy administration in mSTS patients.

Treatment costs included all costs incurred on eligible IV cancer therapy dates of service. Costs were categorized as (i) IV cancer therapy administration procedure costs, (ii) IV cancer therapy drug costs for mSTS, and (iii) costs associated with other visit-related drugs and services provided on the day of the IV cancer therapy visit. Claim codes used to identify IV infusion administration costs are consistent with those used in previous research [8–10].

Treatment costs were calculated per patient per IV visit (PPPV) and per patient per month (PPPM). PPPM costs were calculated by first computing average per month treatment costs for each patient, defined as the sum of all costs on days with IV cancer therapy administrations during the observation period divided by the length of the observation period for each patient in person-months. Average PPPM treatment costs for the overall sample were then calculated as a weighted average of the average per month cost, using the length of the observation period as the weight. Reporting PPPM costs is an approach commonly used in nonexperimental study settings to account for different lengths of observation periods among study patients.

### 2.5. Statistical Analyses

Patient characteristics as of the index date were obtained from the Experian dataset. Age was standardized as patient age in 2009 and divided into five categories: less than 25, from 25 to 39, from 40 to 54, from 55 to 64, and greater than 64 years. Insurance type was defined at the time of the index mSTS diagnosis and was held constant during the study period. Patient characteristics were reported as frequencies and percentages for categorical variables and sample size, means, standard deviations, medians, and ranges for continuous variables.

Medians, means, standard deviations, and quartile ranges of PPPV and PPPM treatment costs were reported for the overall mSTS patient sample. The total and component IV administration costs were further stratified by patient cohorts based on age, gender, region, insurance status, IV drugs, and number of administered drugs during the visit. Cost data was adjusted based on the medical consumer price index (CPI) [12] and reported in 2011 in US dollars. Two sensitivity analyses were performed to assess potential biases arising from patient selection and outliers: (i) analyses were repeated based on an alternative definition of mSTS diagnosis in which patients were only required to have at least two medical claims with an ICD-9-CM diagnosis code of 171; (ii) analyses were repeated after excluding IV administration visits with administration costs in the top 1%. Analyses were performed using SAS 9.3 (SAS Institute, Inc., Cary, NC, USA).

## 3. Results

A total of 1,228 patients with mSTS were identified. Baseline patient characteristics are presented in Table 1. The sample included a broad range of the patient age groups with almost a quarter of patients with ages greater than 64 years. Gender was not consistently recorded on all claims; only about half (48%) of patients had gender recorded. The numbers of reported male and female patients in the sample were similar. About half (50.7%) of the patients were insured by managed care, and another quarter (25.0%) were covered by Medicare (either traditional FFS or Medicare HMOs). The South was the most represented region which contained about half (49.7%) of the patient sample, with fewer patients residing in the Northeast (3.3%) and Southwest (8.7%).

### 3.1. PPPV Costs

Per patient per IV visit costs (PPPV) in which an IV cancer therapy was administered, subdivided into IV drug costs, IV administration costs, and other visit-related service costs, are presented in Table 2. The mean (median) cost per IV visit across the entire mSTS patient sample was $2,427 ($1,532). IV administration costs represented 16.5% of these costs with a mean of $399 per visit. About three-quarters (74.5%) of the IV administration costs were associated with direct administration of the cancer therapy (infusion time), with the remaining quarter of costs associated with therapeutic, diagnostic, and prophylactic administration (24.1%) and hydration administration (1.4%).

Mean IV drug costs were $1,450 and represented 59.7% of the total cost of the visit. The remaining 23.8% of the visit costs ($578) were attributed to other visit-related services. Evaluation and management office visits, supplies and equipment, and other miscellaneous administration comprised a small part of these costs with a mean of $22 (3.7% of other visit-related services). The remaining 96.2% of other visit-related costs were other IV drugs and specially administered oral drugs. Drugs to treat the symptoms of the disease and side effects comprised the largest part of these other drugs: colony-stimulating factors mean cost of $160 per visit, antiemetic agents mean cost of $171 per visit, and antihypercalcemic agents mean cost of $30 per visit. The other large cost categories were off-label chemotherapy agents (mean cost of $50 PPPV) and monoclonal antibody agents (mean cost of $84 PPPV).

IV visit costs on a PPPV basis, broken down by age, gender, region, type of insurance, IV drug used to treat mSTS, and number of IV drugs administered per visit, are presented in Table 3(a). IV administration costs ranged from $311 to $457 (13.9% to 28.1% of total IV visit costs) across age groups. Patients with ages less than 25 years and greater than 64 years had the lowest administration costs. IV administration costs and costs of other visit-related services were very similar between females and males. Patients in the Southwest region reported the lowest IV administration costs ($308) and other visit-related services ($341), and patients in the West reported the highest IV administration costs ($489) and other visit-related services ($646).

The largest differences in IV administration costs were found by insurance status. Managed care patients had the highest IV administration costs (mean $504 per visit) while Medicaid patients had the lowest (mean $92 per visit). The most expensive IV drugs in terms of IV administration costs were anthracycline-based therapies at $479 per visit and the cheapest were angiogenesis-based therapies at $301 per visit. Finally, costs of IV administration tended to increase with the number of IV drugs administered in a visit. Administering a single therapy cost an average of $304, increasing to $693 for three therapies and $936 for four therapies.

### 3.2. PPPM Costs

Total mean PPPM costs, subdivided into IV drug costs, IV administration costs, and other visit-related service costs, are presented in Table 2. Total mean (median) PPPM cost for the entire sample was $5,468 ($4,310) with quartile ranges from $2,066 to $7,431. Mean IV administration costs were $900 PPPM, of which $671 was due to chemotherapy administration, $217 was due to therapeutic, diagnostic, and prophylactic administration, and $12 was due to hydration administration. Mean PPPM cost per IV drug was $3,268 with quartile ranges between $427 and $4,704. Other visit-related costs were $1,300 PPPM. Supplies/equipment, evaluation and management office visits, and miscellaneous administration costs accounted for about $50 PPPM. Among other (i.e., non-mSTS) IV drugs administered during the visits, the costliest were those used to control side effects, including antiemetic agents ($384 PPPM) and colony-stimulating factors ($359 PPPM). Other high-cost categories include monoclonal antibody agents ($190 PPPM), off-label chemotherapy ($111 PPPM), and other miscellaneous agents ($113 PPPM).

IV visit costs on a PPPM basis, broken down by age, gender, region, type of insurance, IV drug used to treat mSTS, and number of IV drugs administered per visit, are presented in Table 3(b). Administration costs were similar across most age groups except for patients greater than 64 years, who had average administration costs about two-thirds ($623 PPPM) the size of the other age categories. Similar to the trend in total costs, patients in the Midwest and the West reported much higher IV administration costs PPPM at $1,080 and $1,030, respectively. Patients in the Northeast and Southwest reported much lower other visit-related costs than other regions.

Large variations in PPPM IV administration and other visit-related service costs were observed by insurance status. The highest IV administration PPPM costs were found in managed care patients and indemnity patients at $1,121 and $1,052, respectively. These patients also experienced higher other visit-related service costs compared to other insurance types. As with the PPPV results, other visit-related service costs were highest among patients with workers' compensation, Tricare, or self-pay insurance. In contrast to the PPPV where anthracycline-based agents were reported to have higher IV administration costs, the alkylating-based agents had much higher PPPM IV administration costs compared to the other therapies. Finally, the step-like trend of increasing IV administration costs based on the number of administered IV drugs during the visit was also observed in the PPPM analysis.

### 3.3. Sensitivity Analyses

Tables 4(a) and 4(b) report the results of two sensitivity analyses undertaken to assess the extent of any potential biases in the results arising from patient selection and outliers. In Table 4(a), total and subdivided PPPV costs were based on an alternative definition of mSTS diagnosis in which patients were only required to have at least two medical claims with an ICD-9-CM diagnosis code of 171. IV administration costs were generally similar and in fact represented a slightly larger percentage of total IV visit costs (17.1%), compared to those based on the primary definition of mSTS diagnosis presented in Table 3(a).

In Table 4(b), total and subdivided PPPV costs are reported after excluding visits with administration costs in the top 1%. Again, results were generally similar to those based on all IV administration visits, presented in Table 3(a). In particular, IV administration costs represented 15.9% of total IV visit costs.

## 4. Discussion

This retrospective analysis assessed costs associated with the administration of IV cancer therapies in mSTS patients from 2005 to 2012 using Experian data. IV administration costs accounted for about 16.5% of costs per IV visit among patients with mSTS. Other visit-related service costs accounted for about 23.8% of the cost PPPV. These results indicate that non-IV drug costs represent a considerable proportion of the total costs when receiving an IV cancer therapy to treat mSTS.

Cost estimates stratified by patient characteristics indicate that some of the largest differences in IV administration costs across patients exist between patients with different types of insurance. Patients with managed care had the highest IV administration costs ($504 PPPV), while Medicaid patients had the lowest ($92 PPPV). IV administration costs also varied widely across regions, as well as by the type of IV cancer drug and number of IV cancer drugs administered.

The share of total treatment costs related to IV administration reported in this study of mSTS patients is similar to estimates reported in both malignant and nonmalignant diseases where drugs are administered by IV infusion. A 2008 study of treatment costs in patients with metastatic breast cancer reported average total treatment costs of $2,477 PPPV, 10.2% of which were IV administration costs [9]. In a separate 2008 study of patients with small cell lung cancer, IV administration costs were estimated to be 11.8% of average total costs [8]. Finally, in a 2011 study of costs in rheumatoid arthritis patients, IV administration costs were 7.9% of average total costs [10].

This study did not account for indirect costs, such as those associated with patient and caregiver time (e.g., travel time) and lost productivity, which by their nature are more difficult to collect and suffer from greater variability. By only examining direct healthcare-system-related costs of IV cancer therapy administration in an mSTS sample, this study likely understates the true total cost of IV therapy to society.

As with all retrospective studies using claims data, identification of mSTS patients relies on the accuracy of diagnosis coding. No clinical information was available to ascertain stage. Advanced STS was identified based on a claims algorithm that has not been validated. To address this concern, a sensitivity analysis was performed where costs were calculated using an alternative claims algorithm. Results of this sensitivity analysis were similar to those reported here. In addition, costs related to IV cancer therapy administration were determined only from clinic claims and were limited to services identified on the claim as an infusion administration cost. This method may underestimate true medical infusion costs since it does not capture costs related to late infusion reactions and complications that require medical care on days following the infusion. Clinic claims also exclude any additional costs of oral drugs or drugs administered in an inpatient setting.

Other study limitations included limited information about patient enrollment and disease progression. Patients appear in the dataset through clinic visits, but the date of initial diagnosis of mSTS may have occurred earlier. In addition, it was not possible to determine why patients may have stopped visiting the clinic. It was also not possible to assess disease progression. To address these limitations, costs were estimated on the basis of actual clinic visits and reported both PPPV and PPPM.

## 5. Conclusion

Using data from the Experian database, this study found that the mean cost of receiving IV cancer therapy in an mSTS patient population was $2,427 PPPV and $5,468 PPPM. IV administration costs accounted for 16.5% of these costs and other visit-related services accounted for 23.8% of these costs. Thus, nonstudy drug related costs are about 40% of the costs associated with administration of IV cancer therapy for mSTS patients. There was substantial variation in the cost of IV administration by insurance type, region, type of anticancer drug, and the number of anticancer drugs administered in a single visit.

This study contributes to the understanding of IV therapy administration costs in mSTS patients. Further research is needed to investigate the association between IV administration costs and mSTS cancer stage and to estimate indirect costs of IV administration associated with mSTS patient and caregiver time.

## Figures and Tables

**Table 1:** Patient characteristics for mSTS sample.

Patient characteristic	Count (*N* = 1,228)	Percent
Age^1^		
Less than 25	117	9.5%
25 to 39	152	12.4%
40 to 54	316	25.7%
55 to 64	345	28.1%
Greater than 64	298	24.3%
Gender^2^		
Female	289	23.5%
Male	295	24.0%
Insurance type^3^		
Managed care	622	50.7%
Medicare	283	23.0%
Medicare HMO	24	2.0%
Medicaid	67	5.5%
Medicaid HMO	10	0.8%
Indemnity	68	5.5%
Other^4^	154	12.5%
Region		
Midwest	240	19.5%
Northeast	41	3.3%
South	610	49.7%
Southwest	107	8.7%
West	230	18.7%

1: Age of the patient in 2009.

2: 644 patients were missing gender information.

3: Insurance type is defined at the time of the index mSTS diagnosis.

4: Other includes workers' compensation, Tricare, and self-pay.

**Table 2:** IV visit costs per patient per visit (PPPV) and per patient per month (PPPM) by cost categories.

No. of visits												
PPPV	13,583											
PPPM	6,025											
			Costs PPPV ($)^1^					Costs PPPM ($)^1^				
Cost category	Sum ($)^2^	%	Median	Mean	Std. dev.	1st quartile	3rd quartile	Median	Mean	Std. dev.	1st quartile	3rd quartile
Total	**32,970,401**	**100.0%**	**1,532.11**	**2,427.33**	**2,674.95**	**720.50**	**3,187.80**	**4,310.20**	**5,467.61**	**133.01**	**2,065.64**	**7,430.99**
IV administration	**5,425,305**	**16.5%**	**342.01**	**399.42**	**291.78**	**226.62**	**513.84**	**678.54**	**899.57**	**24.91**	**395.94**	**1,130.75**
Chemotherapy administration	4,044,094	74.5%	253.53	297.73	227.91	163.67	379.00	489.72	670.50	19.34	282.65	832.90
Hydration administration	75,244	1.4%	0.00	5.54	23.55	0.00	0.00	0.00	12.45	1.26	0.00	2.55
Therapeutic, diagnostic, prophylactic admin.	1,305,967	24.1%	77.40	96.15	100.63	31.94	130.82	156.15	216.61	7.12	72.72	276.57
IV drug (for mSTS)	**19,693,317**	**59.7%**	**537.77**	**1,449.85**	**2,175.31**	**98.59**	**1,828.55**	**2,236.07**	**3,268.45**	**102.50**	**427.21**	**4,703.56**
Other visit-related services	**7,851,778**	**23.8%**	**190.55**	**578.06**	**1,178.00**	**42.31**	**483.00**	**725.44**	**1,299.60**	**51.60**	**294.02**	**1,573.49**
Miscellaneous administration	112,606	1.4%	0.00	8.29	72.83	0.00	0.00	0.00	18.68	1.52	0.00	15.69
Supplies/equipment	14,537	0.2%	0.00	1.07	13.76	0.00	0.00	0.00	2.39	0.57	0.00	0.00
Office visit/evaluation and mgmt. services	168,391	2.1%	0.00	12.40	41.77	0.00	0.00	0.00	27.90	2.00	0.00	4.18
Other IV drugs/specially admin. oral drugs	7,556,244	96.2%	185.76	556.30	1,167.77	17.25	468.02	688.65	1,250.63	51.33	241.48	1,498.94
Antihypercalcemic agents	407,395	5.4%	0.00	29.99	260.40	0.00	0.00	0.00	67.62	7.25	0.00	0.00
Colony-stimulating factor	2,175,927	28.8%	0.00	160.19	699.58	0.00	0.00	0.00	358.66	27.14	0.00	58.14
Antiemetic	2,317,729	30.7%	95.85	170.63	220.46	0.71	254.66	291.67	384.22	11.32	54.81	556.62
Use for IV infusion	90,036	1.2%	0.00	6.63	15.56	0.00	2.72	0.21	14.94	1.25	0.00	5.39
Corticosteroid for hypersensitivity	53,693	0.7%	1.09	3.95	16.74	0.00	2.33	2.67	8.91	0.91	0.98	6.32
Used to clear (flush) IV lines or catheters	6,515	0.1%	0.00	0.48	5.49	0.00	0.00	0.00	1.08	0.39	0.00	0.00
H2 antagonists for hypersensitivity	3,533	0.0%	0.00	0.26	2.54	0.00	0.00	0.00	0.59	0.08	0.00	0.00
For anemia	4,987	0.1%	0.00	0.37	7.79	0.00	0.00	0.00	0.83	0.23	0.00	0.00
Anticancer agent monoclonal antibody	1,143,295	15.1%	0.00	84.17	713.08	0.00	0.00	0.00	189.76	32.30	0.00	0.00
Anticancer agent chemotherapy	674,420	8.9%	0.00	49.65	319.96	0.00	0.00	0.00	111.43	21.30	0.00	0.00
Misc. other drugs	678,714	9.0%	0.00	49.97	358.51	0.00	1.34	1.93	112.59	10.55	0.00	26.98

1: Treatment costs derived from the contracted allowed payment for a claim.

2: Sum over all visits/months and patients.

**Table:** (a)

Category	No. of patients	No. of visits	Total		IV drug		IV administration		Other visit-related services	
			Average costs ($)	%	Average costs ($)	%	Average costs ($)	%	Average costs ($)	%
Total	**1,228**	**13,583**	**2,427.33**	**100.0%**	**1,449.85**	**59.7%**	**399.42**	**16.5%**	**578.06**	**23.8%**
Age										
Less than 25	117	1,944	1,117.32	100.0%	462.60	41.4%	314.53	28.1%	340.19	30.4%
25 to 39	152	2,001	2,228.42	100.0%	1,276.28	57.3%	447.07	20.1%	505.07	22.7%
40 to 54	316	3,471	2,632.44	100.0%	1,531.81	58.2%	435.10	16.5%	665.52	25.3%
55 to 64	345	3,351	3,292.29	100.0%	2,057.09	62.5%	457.14	13.9%	778.06	23.6%
Greater than 64	298	2,816	2,190.91	100.0%	1,431.09	65.3%	311.49	14.2%	448.33	20.5%
Gender^2^										
Female	289	3,367	2,149.91	100.0%	1,282.05	59.6%	341.70	15.9%	526.16	24.5%
Male	295	3,617	2,082.48	100.0%	1,162.35	55.8%	360.57	17.3%	559.56	26.9%
Region										
Midwest	240	2,653	2,471.90	100.0%	1,485.45	60.1%	424.76	17.2%	561.69	22.7%
Northeast	41	342	2,240.14	100.0%	1,348.44	60.2%	391.31	17.5%	500.39	22.3%
South	610	7,112	2,140.51	100.0%	1,166.50	54.5%	373.68	17.5%	600.33	28.0%
Southwest	107	1,064	1,675.80	100.0%	1,026.85	61.3%	308.40	18.4%	340.56	20.3%
West	230	2,412	3,582.08	100.0%	2,447.14	68.3%	488.75	13.6%	646.19	18.0%
Insurance type^3^										
Managed care	622	6,874	2,936.17	100.0%	1,783.86	60.8%	504.22	17.2%	648.10	22.1%
Medicare	283	2,763	1,879.52	100.0%	1,182.80	62.9%	271.84	14.5%	424.88	22.6%
Medicare HMO	24	251	1,543.22	100.0%	880.70	57.1%	241.51	15.6%	421.01	27.3%
Medicaid	67	787	859.31	100.0%	344.74	40.1%	91.61	10.7%	422.96	49.2%
Medicaid HMO	10	145	709.35	100.0%	334.71	47.2%	185.94	26.2%	188.70	26.6%
Indemnity	68	1,036	2,199.25	100.0%	1,359.22	61.8%	371.24	16.9%	468.80	21.3%
Other^4^	154	1,727	2,402.50	100.0%	1,281.98	53.4%	384.41	16.0%	736.11	30.6%
IV drug (for mSTS)^5^										
Gemcitabine-based	429	3,315	3,302.95	100.0%	2,447.16	74.1%	358.70	10.9%	497.09	15.0%
Anthracycline-based	434	2,005	2,344.63	100.0%	1,024.02	43.7%	479.23	20.4%	841.38	35.9%
Alkylating-agents-based	424	3,772	1,469.59	100.0%	474.53	32.3%	433.81	29.5%	561.25	38.2%
Angiogenesis inhibitors	58	374	5,249.94	100.0%	4,291.45	81.7%	301.03	5.7%	657.47	12.5%
Taxane-based	172	1,123	2,184.18	100.0%	1,323.34	60.6%	409.22	18.7%	451.62	20.7%
Other	352	2,994	2,458.42	100.0%	1,552.03	63.1%	356.33	14.5%	550.06	22.4%
Number of administered IV drugs^6^										
One	998	8,016	1,855.27	100.0%	1,007.91	54.3%	304.23	16.4%	543.13	29.3%
Two	680	4,336	3,423.98	100.0%	2,348.01	68.6%	481.76	14.1%	594.21	17.4%
Three	161	1,049	2,515.14	100.0%	1,058.61	42.1%	693.36	27.6%	763.17	30.3%
Four	47	182	3,372.39	100.0%	1,771.78	52.5%	935.96	27.8%	664.65	19.7%

**Table:** (b)

Category	No. of patients	No. of months	Total		IV drug		IV administration		Other visit-related services	
			Average costs ($)	%	Average costs ($)	%	Average costs ($)	%	Average costs ($)	%
Total	**1,228**	**6,025.0**	**5,467.61**	**100.0%**	**3,268.45**	**59.8%**	**899.57**	**16.5%**	**1,299.60**	**23.8%**
Age										
Less than 25	117	623.3	3,476.65	100.0%	1,442.50	41.5%	978.12	28.1%	1,056.03	30.4%
25 to 39	152	836.2	5,338.35	100.0%	3,054.94	57.2%	1,070.62	20.1%	1,212.80	22.7%
40 to 54	316	1,513.3	6,030.91	100.0%	3,511.16	58.2%	997.38	16.5%	1,522.37	25.2%
55 to 64	345	1,650.8	6,686.45	100.0%	4,181.94	62.5%	928.28	13.9%	1,576.23	23.6%
Greater than 64	298	1,401.4	4,386.23	100.0%	2,869.85	65.4%	623.09	14.2%	893.29	20.4%
Gender^2^										
Female	289	1,588.8	4,544.31	100.0%	2,712.88	59.7%	721.32	15.9%	1,110.11	24.4%
Male	295	1,563.7	4,819.57	100.0%	2,689.15	55.8%	835.49	17.3%	1,294.93	26.9%
Region										
Midwest	240	1,040.6	6,289.83	100.0%	3,784.40	60.2%	1,079.88	17.2%	1,425.55	22.7%
Northeast	41	207.8	3,687.44	100.0%	2,219.64	60.2%	644.12	17.5%	823.67	22.3%
South	610	3,200.9	4,753.24	100.0%	2,590.95	54.5%	830.59	17.5%	1,331.70	28.0%
Southwest	107	434.6	4,106.94	100.0%	2,531.00	61.6%	754.76	18.4%	821.18	20.0%
West	230	1,141.2	7,563.95	100.0%	5,170.11	68.4%	1,030.27	13.6%	1,363.58	18.0%
Insurance type^3^										
Managed care	622	3,088.9	6,532.36	100.0%	3,971.37	60.8%	1,120.58	17.2%	1,440.41	22.1%
Medicare	283	1,374.0	3,764.41	100.0%	2,374.73	63.1%	544.94	14.5%	844.73	22.4%
Medicare HMO	24	113.4	3,415.78	100.0%	1,949.36	57.1%	534.56	15.6%	931.86	27.3%
Medicaid	67	375.7	1,804.71	100.0%	722.61	40.0%	196.05	10.9%	886.05	49.1%
Medicaid HMO	10	37.9	2,716.27	100.0%	1,281.66	47.2%	712.02	26.2%	722.59	26.6%
Indemnity	68	365.1	6,232.59	100.0%	3,853.04	61.8%	1,051.79	16.9%	1,327.76	21.3%
Other^4^	154	670.0	6,191.80	100.0%	3,305.31	53.4%	991.86	16.0%	1,894.63	30.6%
IV drug (for mSTS)										
Gemcitabine-based	429	1,577.9	7,021.77	100.0%	5,208.74	74.2%	761.17	10.8%	1,051.86	15.0%
Anthracycline-based	434	1,243.0	3,752.46	100.0%	1,629.15	43.4%	768.24	20.5%	1,355.07	36.1%
Alkylating-agents-based	424	1,221.5	4,753.73	100.0%	1,559.65	32.8%	1,365.87	28.7%	1,828.21	38.5%
Angiogenesis inhibitors	58	321.6	7,532.36	100.0%	6,191.35	82.2%	460.33	6.1%	880.67	11.7%
Taxane-based	172	575.0	4,253.79	100.0%	2,563.94	60.3%	802.76	18.9%	887.08	20.9%
Other^5^	352	1,086.0	6,006.84	100.0%	3,755.13	62.5%	907.76	15.1%	1,343.95	22.4%
Number of administered IV drugs^6^										
One	998	2,492.2	3,950.35	100.0%	2,105.69	53.3%	610.28	15.4%	1,234.38	31.2%
Two	680	2,781.1	6,616.14	100.0%	4,463.78	67.5%	954.84	14.4%	1,197.52	18.1%
Three	161	514.1	6,725.12	100.0%	2,888.23	42.9%	1,631.79	24.3%	2,205.10	32.8%
Four	47	237.6	5,218.13	100.0%	2,296.07	44.0%	1,702.72	32.6%	1,219.34	23.4%

1: Treatment costs derived from the contracted allowed payment for a claim.

2: 644 patients were missing gender information.

3: Insurance type is defined at the time of the index mSTS diagnosis.

4: Other includes workers' compensation, Tricare, and self-pay.

5: Gemcitabine-based includes gemcitabine monotherapy and combination therapies. Anthracycline-based includes doxorubicin and epirubicin monotherapies and combination therapies. Alkylating-agents-based includes monotherapies and combination therapies with cisplatin, cyclophosphamide, dacarbazine, etoposide, ifosfamide, temozolomide, and topotecan. Angiogenesis inhibitors include bevacizumab monotherapy. Taxane-based includes docetaxel and paclitaxel monotherapies and combination therapies. Other includes actinomycin D, carboplatin, irinotecan, interferon, oxaliplatin, vincristine, and vinorelbine.

6: Number of mSTS treatments that were administered during the visit.

**Table:** (a)

Category	No. of patients	No. of visits	Total		IV drug		IV administration		Other visit-related services	
			Average costs ($)	%	Average costs ($)	%	Average costs ($)	%	Average costs ($)	%
Total	**1,023**	**11,777**	**2,293.60**	**100.0%**	**1,334.09**	**58.2%**	**392.81**	**17.1%**	**566.71**	**24.7%**
Age										
Less than 25	112	1,872	1,067.78	100.0%	435.96	40.8%	304.44	28.5%	327.38	30.7%
25 to 39	138	1,897	2,077.10	100.0%	1,153.72	55.5%	439.82	21.2%	483.56	23.3%
40 to 54	266	2,999	2,569.69	100.0%	1,486.01	57.8%	442.27	17.2%	641.41	25.0%
55 to 64	282	2,833	3,159.29	100.0%	1,941.58	61.5%	438.70	13.9%	779.01	24.7%
Greater than 64	225	2,176	2,029.35	100.0%	1,263.70	62.3%	299.93	14.8%	465.72	22.9%
Gender^2^										
Female	234	2,882	1,971.97	100.0%	1,135.12	57.6%	332.27	16.8%	504.58	25.6%
Male	234	3,156	1,856.48	100.0%	970.27	52.3%	347.30	18.7%	538.91	29.0%
Region										
Midwest	189	2,235	2,206.58	100.0%	1,292.76	58.6%	423.45	19.2%	490.38	22.2%
Northeast	19	199	2,733.61	100.0%	1,665.07	60.9%	367.43	13.4%	701.11	25.6%
South	513	6,204	2,001.71	100.0%	1,021.93	51.1%	367.81	18.4%	611.97	30.6%
Southwest	95	985	1,620.03	100.0%	1,021.50	63.1%	308.51	19.0%	290.02	17.9%
West	207	2,154	3,491.98	100.0%	2,388.44	68.4%	473.90	13.6%	629.64	18.0%
Insurance type^3^										
Managed care	530	6,076	2,773.41	100.0%	1,646.46	59.4%	492.05	17.7%	634.91	22.9%
Medicare	217	2,200	1,756.68	100.0%	1,073.70	61.1%	271.13	15.4%	411.86	23.4%
Medicare HMO	19	181	1,808.04	100.0%	959.11	53.0%	274.88	15.2%	574.05	31.7%
Medicaid	61	756	814.99	100.0%	282.49	34.7%	93.08	11.4%	439.42	53.9%
Medicaid HMO	9	141	614.82	100.0%	233.36	38.0%	187.46	30.5%	193.99	31.6%
Indemnity	55	881	2,003.24	100.0%	1,200.97	60.0%	373.60	18.6%	428.67	21.4%
Other^4^	132	1,542	2,270.35	100.0%	1,211.04	53.3%	365.92	16.1%	693.39	30.5%
IV drug (for mSTS)^5^										
Gemcitabine-based	374	2,926	3,438.08	100.0%	2,551.62	74.2%	366.02	10.6%	520.44	15.1%
Anthracycline-based	406	1,904	2,304.97	100.0%	1,006.55	43.7%	483.34	21.0%	815.08	35.4%
Alkylating-agents-based	383	3,416	1,494.70	100.0%	498.44	33.3%	430.76	28.8%	565.51	37.8%
Angiogenesis inhibitors	33	250	5,499.09	100.0%	4,413.94	80.3%	276.56	5.0%	808.59	14.7%
Taxane-based	141	945	2,280.97	100.0%	1,371.47	60.1%	416.92	18.3%	492.59	21.6%
Other	260	2,336	1,681.11	100.0%	953.29	56.7%	299.76	17.8%	428.06	25.5%
Number of administered IV drugs^6^										
One	831	6,874	1,717.75	100.0%	890.29	51.8%	295.14	17.2%	532.32	31.0%
Two	581	3,779	3,245.44	100.0%	2,178.48	67.1%	467.91	14.4%	599.05	18.5%
Three	148	963	2,449.85	100.0%	1,097.77	44.8%	696.15	28.4%	655.92	26.8%
Four	44	161	3,603.77	100.0%	1,876.43	52.1%	985.49	27.3%	741.85	20.6%

1: mSTS diagnosis defined as at least two medical claims with an ICD-9-CM diagnosis code of 171 and at least one medical claims with a diagnosis of a distant secondary malignant neoplasm (ICD-9-CM 196.XX-199.0). Treatment costs derived from the contracted allowed payment for a claim.

2: 644 patients were missing gender information.

3: Insurance type is defined at the time of the index mSTS diagnosis.

4: Other includes workers' compensation, Tricare, and self-pay.

5: Gemcitabine-based includes gemcitabine monotherapy and combination therapies. Anthracycline-based includes doxorubicin and epirubicin monotherapies and combination therapies. Alkylating-agents-based includes monotherapies and combination therapies with cisplatin, cyclophosphamide, dacarbazine, etoposide, ifosfamide, temozolomide, and topotecan. Angiogenesis inhibitors include bevacizumab monotherapy. Taxane-based includes docetaxel and paclitaxel monotherapies and combination therapies. Other includes actinomycin D, carboplatin, irinotecan, interferon, oxaliplatin, vincristine, and vinorelbine.

6: Number of mSTS treatments that were administered during the visit.

**Table:** (b)

Category	No. of patients	No. of visits	Total		IV drug		IV administration		Other visit-related services	
			Average costs ($)	%	Average costs ($)	%	Average costs ($)	%	Average costs ($)	%
Total	**1,221**	**13,325**	**2,363.53**	**100.0%**	**1,417.87**	**60.0%**	**376.94**	**15.9%**	**568.72**	**24.1%**
Age										
Less than 25	116	1,934	1,104.42	100.0%	459.15	41.6%	307.88	27.9%	337.38	30.5%
25 to 39	151	1,953	2,170.22	100.0%	1,251.49	57.7%	423.35	19.5%	495.38	22.8%
40 to 54	313	3,377	2,565.44	100.0%	1,521.19	59.3%	402.53	15.7%	641.72	25.0%
55 to 64	344	3,274	3,227.77	100.0%	2,021.82	62.6%	429.45	13.3%	776.50	24.1%
Greater than 64	297	2,787	2,112.86	100.0%	1,365.08	64.6%	299.66	14.2%	448.12	21.2%
Gender^2^										
Female	289	3,352	2,143.16	100.0%	1,278.43	59.7%	337.16	15.7%	527.57	24.6%
Male	295	3,604	2,073.06	100.0%	1,158.90	55.9%	357.01	17.2%	557.15	26.9%
Region										
Midwest	240	2,636	2,454.05	100.0%	1,478.01	60.2%	417.56	17.0%	558.48	22.8%
Northeast	41	339	2,242.53	100.0%	1,357.27	60.5%	383.64	17.1%	501.61	22.4%
South	604	6,969	2,084.97	100.0%	1,150.36	55.2%	347.76	16.7%	586.85	28.1%
Southwest	107	1,062	1,669.85	100.0%	1,023.82	61.3%	306.28	18.3%	339.75	20.3%
West	229	2,319	3,433.15	100.0%	2,342.75	68.2%	449.85	13.1%	640.56	18.7%
Insurance type^3^										
Managed care	615	6,647	2,838.48	100.0%	1,737.24	61.2%	468.51	16.5%	632.73	22.3%
Medicare	283	2,759	1,875.14	100.0%	1,180.06	62.9%	270.15	14.4%	424.93	22.7%
Medicare HMO	24	251	1,543.22	100.0%	880.70	57.1%	241.51	15.6%	421.01	27.3%
Medicaid	67	787	859.31	100.0%	344.74	40.1%	91.61	10.7%	422.96	49.2%
Medicaid HMO	10	145	709.35	100.0%	334.71	47.2%	185.94	26.2%	188.70	26.6%
Indemnity	68	1,036	2,199.25	100.0%	1,359.22	61.8%	371.24	16.9%	468.80	21.3%
Other^4^	154	1,700	2,357.83	100.0%	1,259.37	53.4%	364.05	15.4%	734.42	31.1%
IV drug (for mSTS)^5^										
Gemcitabine-based	429	3,305	3,290.15	100.0%	2,439.82	74.2%	354.74	10.8%	495.59	15.1%
Anthracycline-based	423	1,925	2,275.39	100.0%	1,022.92	45.0%	432.97	19.0%	819.50	36.0%
Alkylating-agents-based	419	3,686	1,396.09	100.0%	441.77	31.6%	405.93	29.1%	548.39	39.3%
Angiogenesis inhibitors	58	367	5,217.74	100.0%	4,275.64	81.9%	281.20	5.4%	660.90	12.7%
Taxane-based	172	1,106	2,141.45	100.0%	1,300.21	60.7%	392.87	18.3%	448.37	20.9%
Other	348	2,936	2,319.72	100.0%	1,439.00	62.0%	334.77	14.4%	545.96	23.5%
Number of administered IV drugs^6^										
One	997	7,994	1,842.21	100.0%	998.14	54.2%	300.77	16.3%	543.30	29.5%
Two	670	4,229	3,333.67	100.0%	2,291.94	68.8%	456.61	13.7%	585.11	17.6%
Three	156	966	2,353.14	100.0%	1,023.01	43.5%	613.09	26.1%	717.04	30.5%
Four	39	136	2,913.93	100.0%	1,714.22	58.8%	699.57	24.0%	500.14	17.2%

1: Treatment costs derived from the contracted allowed payment for a claim.

2: 644 patients were missing gender information.

3: Insurance type is defined at the time of the index mSTS diagnosis.

4: Other includes workers' compensation, Tricare, and self-pay.

5: Gemcitabine-based includes gemcitabine monotherapy and combination therapies. Anthracycline-based includes doxorubicin and epirubicin monotherapies and combination therapies. Alkylating-agents-based includes monotherapies and combination therapies with cisplatin, cyclophosphamide, dacarbazine, etoposide, ifosfamide, temozolomide, and topotecan. Angiogenesis inhibitors include bevacizumab monotherapy. Taxane-based includes docetaxel and paclitaxel monotherapies and combination therapies. Other includes actinomycin D, carboplatin, irinotecan, interferon, oxaliplatin, vincristine, and vinorelbine.

6: Number of mSTS treatments that were administered during the visit.
